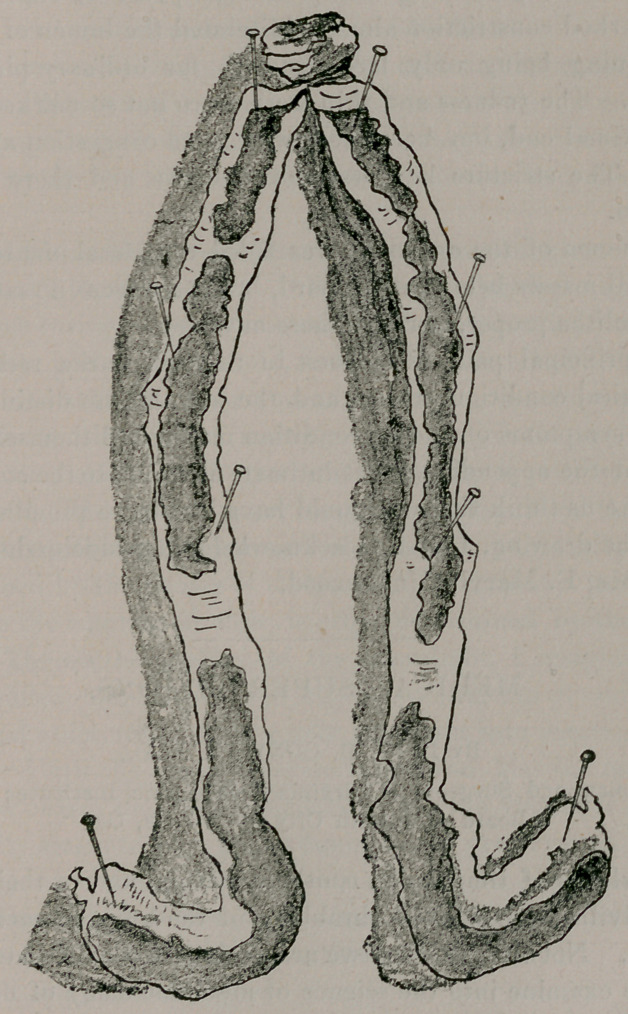# An Interesting Case of Appendicitis

**Published:** 1899-09

**Authors:** W. Hal Moncrief

**Affiliations:** First Assistant House Staff, Grady Hospital, Atlanta, Ga.


### AN INTERESTING CASE OF APPENDICITIS.

By W. HAL MONCRIEF, M.D.,
First Assistant House Staff, Grady Hospital, Atlanta.

Trusting that it will prove of interest to the medical profession of Georgia, I will report the case of appendicitis occurring in Dr. Floyd W. McRae, of this city, the operation for which I had the pleasure of witnessing at Dr. Wm. T. Bull's sanitarium, East 33d street, New York City, on May the 13th.

He had an attack of typhoid fever the latter part of August, 1897—a typical case, with practically all the classical symptoms except hemorrhage, and fever lasted just twenty-one days.

Convalescence from this attack was rapid and uneventful, and, to all appearances, complete.

In the spring of 1898, however, he began to have a little discomfort in the right iliac region, but no well-defined tenderness or localized pain. This lasted for several days, gradually wearing off, but returned at irregular and indefinite intervals, never accompanied by any elevation of pulse or temperature.

On the night of April 26, 1899, he went to bed feeling perfectly well, but awoke about 3 a.m. with headache, nausea and general abdominal distress. On getting up and going to the bathroom he had a distinct chill, and this was followed by considerable fever. He took an antipyretic to relieve headache, and soon went to sleep. Got up about 10 a. m. and went to office, but felt quite uncomfortable, having passed neither feces nor flatus—something very unusual, as his bowels were habitually very regular, moving every morning. Temperature, taken then, 100° F., pulse not taken.

Pain soon became localized in right iliac region, and there was well-marked tenderness over the McBurney point. He took one grain of calomel and followed it by seidlitz powder, and it acted fairly well in the usual time.

The next morning, 28th, pain and tenderness persisted but was somewhat lessened, and temperature $99\frac{2}{5}$° F.

With quiet, rest in bed, liquid diet and free purgation, the symptoms gradually subsided, and by the end of a week there was no discomfort whatever in a recumbent posture, but tenderness on pressure persisted; and after standing or sitting for a length of time he was conscious of an uncomfortable, dull, aching feeling in region of appendix.

This slight discomfort persisting, on his arrival in New York in May he requested Dr. Bull to examine him, which he did, together with Drs. Gibney and Todd; and the result of this examination was the unqualified advice to have the appendix removed.

At this time the tenderness was slightly enhanced and an elongated area of induration, corresponding to the appendix, obtained on palpation.

He accordingly went to Dr. Bull's sanitarium Friday afternoon, May 12, and was prepared for operation on the following day.

The operation started at 9:20 Saturday morning, Dr. Bull operating, assisted by Drs. Walker and Denton. Dr. W. B. Coley and Dr. V. P. Gibney, of New York, with Dr. J. S. Todd, of Atlanta, were present at the operation.

A three-inch incision was made over McBurney's point, rather higher than usual, palpation demonstrating the appendix in that situation. The fibers of the muscles were separated, according to the method of McBurney, and the cecum came into view as soon as the peritoneum was divided. The appendix was easily found, being slightly thickened and indurated, passing backward, inward, and upward behind the cecum.

The tumor-like mass at first palpated was found to have been the thickened cecum wall which covered the appendix.

Sponges were packed around base of appendix to protect abdominal cavity, and the appendix was then ligated with No. 2 catgut about three-eighths of an inch from cecum, another ligature being passed half an inch from first. The appendix was then severed between the two ligatures; and the proximal end buried in a fold of cecum and secured by five sutures of medium-fine black silk.

The distal portion, which was adherent all the way, was then detached from cecum by scissors and blunt dissection, the oozing being stopped by forceps and ligatures of fine catgut. End of appendix was rather bulbous and buried in adhesions.

It extended so high up, being above level of umbilicus, that

Dr. Bull thought he would have to enlarge opening; but, after tedious efforts, he succeeded in raising it from its bed of adhesions and removing it.

There was quite a little oozing from the site of these adhesions, and not wishing to drain if he could avoid it, Dr. Bull packed gauze into the cavity and waited for oozing to stop.

It did not, however, and an iodoform gauze drain was introduced and brought out at upper and outer angle of wound.

The abdominal wall was closed in layers, according to the method practiced by Drs. Bull and Coley; the peritoneum muscle and fascia being brought together with interrupted sutures of fine catgut, the skin being closed with silkworm-gut, which also passed through muscular layer.

A sterile dressing was then applied, one-sixtieth grain strychnine and five minims of Magendie's solution of morphia given, and he was put to bed.

The time of the operation was fifty minutes, it taking five minutes to get in and find the appendix and five to close up; the remainder of the time largely taken up in breaking up the adhesions.

He stood the operation well, had a pulse of 96 when it was finished, and had no trouble with the ether, which was preceded by nitrous oxide. He vomited several times and suffered a good deal of pain and discomfort during the afternoon and evening, and he had to be catheterized for the first twenty-four hours. Though the pain and gas in abdomen were very distressing, he slept some during the night, and at 5 a. m. retained one ounce of coffee.

At 11:30 a. m. the gauze drain was removed, at his urgent request, by Dr. Bull, a few whiffs of gas being administered. The dressings were fairly well soiled, but with nothing but serum. The edges of the wound fell together nicely on removing the drain, and a moist dressing (saline) was then applied. These were changed daily and it healed rapidly, there being no oozing after the third day.

On the 19th two of the three sutures were cut but not removed, working loose gradually, and being entirely free on the dressing the next morning, when the last one was treated in a similar manner.

On the 21st the wound was found entirely healed and union apparently more *per primam* at the site of drainage than at other part of incision.

Permission was given to sit up on the 24th, and he left the sanitarium for the hotel on the 26th, where he remained in his room for a week, getting about gradually.

His pulse never went above 96—which point it reached immediately after the operation. Temperature ranged as high as $99\frac{2}{5}°$ F. for three days succeeding operation, but was normal thereafter.

The accompanying drawing will show the appendix divided from end to end, and to some extent give an idea of the condition present.

The lining of the tube had an ulcerated appearance throughout, but this was specially marked from tip to the largest constriction, the walls at this portion resembling very thin paper, and at convexity of curve, seeming almost at the point of rupture. The most marked constriction almost obliterated the lumen of the tube, the opening being only large enough for ordinary pin to pass through. The redness and thin walls were not so marked towards the proximal end, but here there was more congestion and thickening. The stricture here was not so dense and there was little occlusion.

The lumen of the appendix was filled with fecal matter, one or two small masses being rather hard, but there were no concretions or enteroliths proper, nor was there any pus.

The principal point of interest in the case is the rather grave pathological condition present and the fact that no distinctly well-marked symptoms of such a condition manifested themselves. The length of the appendix and its intimate adhesion to the colon would also make us think that we should have had more prominent signs.

For the drawing, I wish to acknowledge my indebtedness to my friend, Mr. E. Marvin Underwood.

## Figures and Tables

**Figure f1:**